# Genetic Counselling Improves the Molecular Characterisation of Dementing Disorders

**DOI:** 10.3390/jpm11060474

**Published:** 2021-05-26

**Authors:** Stefania Zampatti, Michele Ragazzo, Cristina Peconi, Serena Luciano, Stefano Gambardella, Valerio Caputo, Claudia Strafella, Raffaella Cascella, Carlo Caltagirone, Emiliano Giardina

**Affiliations:** 1 Genomic Medicine Laboratory UILDM, IRCCS Fondazione Santa Lucia, 00179 Rome, Italy; s.zampatti@hsantalucia.it (S.Z.); cristinapeconi@gmail.com (C.P.); serenaluciano@hotmail.it (S.L.); claudia.strafella@gmail.com (C.S.); raffaella.cascella@gmail.com (R.C.); 2 Department of Biomedicine and Prevention, Tor Vergata University of Rome, 00133 Rome, Italy; michele.ragazzo@uniroma2.it (M.R.); v.caputo91@gmail.com (V.C.); 3 IRCCS Neuromed, 86077 Pozzilli, Italy; stefano.gambardella@uniurb.it; 4 Department of Biomolecular Sciences, University of Urbino “Carlo Bo”, 61029 Urbino, Italy; 5 Department of Biomedical Sciences, Catholic University Our Lady of Good Counsel, 1000 Tirana, Albania; 6 Department of Clinical and Behavioral Neurology, IRCCS Fondazione Santa Lucia, 00179 Rome, Italy; c.caltagirone@hsantalucia.it

**Keywords:** genetic counselling, dementia, rare genetic variants

## Abstract

Dementing disorders are a complex group of neurodegenerative diseases characterised by different, but often overlapping, pathological pathways. Genetics have been largely associated with the development or the risk to develop dementing diseases. Recent advances in molecular technologies permit analyzing of several genes in a small time, but the interpretation analysis is complicated by several factors: the clinical complexity of neurodegenerative disorders, the frequency of co-morbidities, and the high phenotypic heterogeneity of genetic diseases. Genetic counselling supports the diagnostic path, providing an accurate familial and phenotypic characterisation of patients. In this review, we summarise neurodegenerative dementing disorders and their genetic determinants. Genetic variants and associated phenotypes will be divided into high and low impact, in order to reflect the pathologic continuum between multifactorial and mendelian genetic factors. Moreover, we report a molecular characterisation of genes associated with neurodegenerative disorders with cognitive impairment. In particular, the high frequency of rare coding genetic variants in dementing genes strongly supports the role of geneticists in both, clinical phenotype characterisation and interpretation of genotypic data. The smart application of exome analysis to dementia patients, with a pre-analytical selection on familial, clinical, and instrumental features, improves the diagnostic yield of genetic test, reduces time for diagnosis, and allows a rapid and personalised management of disease.

## 1. Introduction

Dementia is a disorder that impairs the cognitive function. In general, it is a chronic or progressive impairment of cerebral function that determines a complex cognitive decline, frequently associated with mood, and behavioural and personality disorders. Although the incidence of dementia increases with advanced age, it is not an ineluctable disorder in elderly. Generally, the disease affects the elderly (about 5% to 20% of people over 65 years of age), and is a progressive impairment of cognitive function, sometimes with other neurological signs. It is estimated that 47 millions of people worldwide have been affected by dementia, that is one of the main causes of inability [1]. The improvement in healthcare systems determines a progressive lengthening of life expectancy. It is expected that, with the ageing of a population, the dementia incidence will increase every year [2]. A recent study of the Alzheimer Cohorts Consortium showed that in 27 years about the 9% of people over 65 years of age developed dementia, with a similar distribution in men and women. As previously reported, the incidence increased with the progression of age: from 4:1000 per year in 65–69 y old people to 65:1000 per year in 85–89 y old people. [3] Interestingly, the study shows a reduction in dementia incidence of 13% over the years, in Europe and North America. This reduction could be due to the many improvements in early diagnosis and treatment of dementia and prodromic disorders as MCI. Furthermore, a recent meta-analysis confirmed the increasing prevalence of Alzheimer and Vascular dementia with age, with a slight increase in females compared to age-matched males [4].

From a genetic point of view, it is known that dementing disorders are characterised by several genetic influencers. In particular, a familial risk of disease has been recognised in many forms of dementia. For example, in Alzheimer disease, several genes and genetic loci have been identified as correlated to the development of dementia [5]. Recently, the availability of high throughput technologies permits to extend the scenario of genetic variants that can be investigated. As a consequence, many health organisations have focused research efforts to discover clinical and therapeutic improvements genetically determined [6,7]. It is well known that every human disorder recognises genetic and non-genetic causes. The knowledge of genetic factors involved in the development of disorders will permit the application of personalised protocols of disease prevention or treatment. In particular, the variable portion of human DNA can explain many of the known differences between people. Similarly, the environment produces epigenetic modifications of the DNA that can modify the functional activity of genes [8,9]. 

## 2. Genetic Factors Involved in Cognitive Disorders

Cognitive disorders can recognise strong and light genetic factors. This paper will summarise the main genetic causes of adult-onset dementia due to neurodegenerative diseases. As evident, an accurate familial anamnesis and a genetic counselling can help the diagnostic path and reduce the time for the diagnosis. Genetic causes of dementia will be divided in high and low impact, reflecting the clinical occurrence of familial or sporadic disorders.

Generally, cognitive disorders with a big familial recurrence are due to a small number of variants, that are able to determine a high risk of disease [10]. On the contrary, sporadic dementia generally recognises many genetic variants that confer a small risk of disease [11]. The knowledge and the ability to recognise and distinguish the first from the latter makes the difference. In this scenario, it can be recognised a pathologic continuum between multifactorial and mendelian genetic factors. In fact, many times different variants in the same gene can product quite different phenotypes or a differential risk of disease. In clinical practice, the multi-disciplinary approach to the patient permits to evaluate and correctly interpret the genetic influences in the etiology of diseases. In particular, a combined neurological and genetic approach can optimise the diagnostic path.

### 2.1. Phenotypes Due by High Impact Variants or Genes (Mendelian)

To date, many rare neurodegenerative disorders with cognitive impairment have been described. In order to conduct an appropriate differential diagnosis and to clinically recognise these rare disorders, it is important to consider not only the phenotype, but also the family history. In fact, a superficial evaluation of familial anamnesis can determine a misdiagnosis with important consequences for patients and their families. In this Section, clinical, instrumental, and molecular features of genetic dementing disorders are summarised.

#### 2.1.1. CADASIL

Cerebral autosomal dominant arteriopathy with subcortical infarcts and leukoencephalopathy (CADASIL) is a vascular arteriopathy, characterised by many recurrent small strokes that lead to a subcortical dementia [12]. In particular, it is a non-amyloid dementia characterised by a non-atherosclerotic vasculopathy that involves preferentially small arterioles and arteries. Clinically, it can be recognised by some early signs and symptoms of disease. A frequent (but not constant) feature is the migraine, generally with an onset in the second–third decade [13]. The clinical onset of the disorder can vary from the fourth to the sixth decade [14]. CADASIL became manifest when the patient showed dementing disorder, sometimes with psychiatric features (depression, apathy, and personality changes), seizures, and peripheral neuropathy [15]. Many patients show transient ischemic attacks (TIA) and ischemic strokes that anticipate the psychiatric and dementing features [14].

In the diagnostic path, the instrumental evaluation of a patient with a suspect of CADASIL is fundamental. In particular, the cerebral neuroimaging shows leukoencephalopathy with two typical progressive signs of disease: (i) white matter hyperintensities on MRI (typically starting in the anterior temporal lobes, and successively involving frontal and parietal lobes, and external capsule) [16,17], (ii) cerebral microbleeds (without a clear predominant location) [18].

Sometimes, a skin biopsy can support the diagnosis. The electron microscopy of small arterioles shows granular osmiophilic material (GOM) adjacent to vascular smooth muscle cells, but it is an inconsistent feature [19].

On the contrary, the genetic analysis of *NOTCH3* permits the molecular diagnosis in almost all patients [20,21]. *NOTCH3* gene encodes a single pass transmembrane protein with receptor properties. It is largely expressed on vascular smooth muscle cells. After the ligand binding of *NOTCH3* receptor, the intracellular portion moves to the nucleus and activates transcription factors [15]. In particular, it is known that mutations in exons from 2 to 24 of *NOTCH3* modify the encoding of a repeats in the epidermal growth factor (EGFR) [22]. More than 95% of patients with CADASIL carry a mutation in the *NOTCH3* gene, so the sensibility of the genetic analysis is very high. [21] Therefore, the mutational analysis of *NOTCH3* gene is the gold standard for the diagnosis of CADASIL [23].

Furthermore, the known different pathogenic variants in *NOTCH3* domains permit to estimate the penetrance of the disease in the siblings. In fact, it is known that mutations involving 1 to 6 EGFr domains of *NOTCH3* shown typically a full penetrant disorder with a classical CADASIL phenotype. Nonetheless, mutations in 7 to 34 domains are very common in general population (~1:300) and considered as risk factors for a mild form of cerebral small vessel disease [24,25,26]. 

Interestingly, in a large family with CADASIL a heterozygous mutation in *HTRA1* was identified [27]. *HTRA1* is the gene associated to the autosomal recessive form of cerebral arteriopathy with subcortical infarcts and leukoencephalopathy (CARASIL).

#### 2.1.2. CARASIL

Cerebral arteriopathy autosomal recessive with subcortical infarcts and leukoencephalopathy (CARASIL) is a cerebral small vessel disorder characterised by early onset walking disturbance, scalp alopecia, ischemic stroke, mid- to lower-back pain, and progressive cognitive disorder [27].

Biallelic mutations in *HTRA1* were identified as associated to CARASIL. *HTRA1* is a serine peptidase that physiologically interact with TGF-beta. Mutations in *HTRA1* cause an increased TGF-beta activity and expression of TGFB1 in small arteries [28]. Patients with CARASIL show a cerebral disease similar to CADASIL, with typical associated features. CARASIL became manifest before 55 years with slowly progressive dementia, behaviour changes, and walking disturbance with legs spasticity. Non-neurological features include spondylosis deformans (associated to mid- to lower-back pain) and scalp alopecia that starts before third decade [27].

The cerebral MRI shows white matter and external capsule lesions, with a pattern similar to CADASIL. Before onset of symptoms a cerebral leukoaraiosis can be revealed with MRI or TC. After the clinical onset of disease, the cerebral images show: (i) symmetrical hyperintensity of periventricular and deep white matter; (ii) white matter anomalies in temporal lobe, cerebellum, brainstem, cerebellar peduncle, and external capsule in T2-weighted images; (iii) multiple lacunar infarcts in basal ganglia and subcortical white matter; (iv) multiple microbleeds in cerebral cortex, basal ganglia, brainstem, and cerebellum [29].

Genetic confirmation of CARASIL is possible through molecular analysis of *HTRA1* gene. However, considering the rarity of this disorder, a multi-gene panel is preferred. The application of a multigenic sequencing technology permits the identification of mutations not only in *HTRA1* gene, but also in genes involved in disorders to be considered in differential diagnosis of CARASIL (CADASIL, CARASAL, *COL4A1,* and *COL4A2*-related small vessel disease, Fabry disease, *ITM2B*-related dementia, RVCLS- retinal vasculopathy, with cerebral leukoencephalopathy and systemic manifestations).

#### 2.1.3. Rare Syndromes with Phenotype Variability

In the differential diagnosis of brain vascular genetic syndromes, it is important to consider these rare disorders. Sometimes, the pedigree analysis is complicated by phenotypic variability and often reduced penetrance. In particular, the *COL4A1*-related disorders have been associated to different phenotypes (porencephaly; brain small-vessel disease with hemorrhage; angiopathy with nephropathy, aneurysms, and muscle cramps —HANAC-syndrome; tortuosity of retinal arteries; non-syndromic congenital cataract) with a known wide intrafamilial variability. The familial porencephaly is characterised by a wide range of onset (even intrafamilial), with an extreme heterogeneity of manifestations (from infantile hemiparesis to adult-onset migraine) [30]. Sometimes, the MRI shows signs of disease (porencephalic cavities) in asymptomatic patients. Other manifestations associated to *COL4A1* porencephaly are congenital cataract and, less frequently, retinal arteriolar tortuosity. [31]. Moreover, brain small-vessel disease with hemorrhage is a neurodegenerative disorder with clinical signs quite similar to porencephaly (from infantile hemiparesis to migraine with aura or absence of symptoms). The brain MRI shows the characteristic small-vessel disease (periventricular leukoencephalopathy, lacunar infarcts, micro-bleeding, deep intracerebral hemorrhages, and intracerebral calcification) [32]. Even for this phenotype, ocular manifestations have been recorded (congenital cataract, retinal arteriolar tortuosity, and Axenfeld-Rieger anomalies) [31]. Furthermore, systemic involvement can affect muscles (hyperCKemia with or without muscular cramps) and kidneys (hematuria, renal atrophy, renal cysts, and hemolytic anemia) [30].

Hereditary angiopathy with nephropathy, aneurysms, and muscle cramps (HANAC) syndrome is a *COL4A1*-related phenotype with an asymptomatic brain small-vessel disease and frequent systemic manifestations, as muscular cramps, kidney and retinal involvement, and Raynaud phenomenon [30]. The MRI shows leukoencephalopathy with involvement of subcortical, periventricular, or pontine regions, dilated perivascular spaces, lacunar infarcts, and microbleeds. A rare manifestation is the formation of intracranial aneurisms [33].

Although classic Fabry disease has a suggestive phenotype (acroparesthesia, angiokeratomas, sweating abnormalities, corneal and lenticular opacities, and proteinuria), recently cryptogenic stroke has been associated to *GBA* mutations [34]. Consequently, the atypical phenotype of Fabry disease can involve cerebrovascular manifestations that became evident at onset of disorder and even in adulthood. The different phenotype in Fabry disease has been associated with a different activity of the alfa-galattosidase A (α-Gal A). Patients with an α-Gal A activity <1% are more prone to show the typical phenotype of Fabry disease, with onset in infancy. Patients with an α-Gal A activity >1% generally show atypical phenotypes as: (i) late-onset cardiac phenotype (left ventricular hypertrophy, cardiomyopathy, arrhythmia, and proteinuria (without renal insufficiency); (ii) renal variant phenotype (with renal insufficiency, without cutaneous lesions); (iii) cerebrovascular disease (TIA or stroke) [34,35].

Another multisystemic phenotype is associated to mutations in *TREX1* gene, responsible of retinal vasculopathy with cerebral leukoencephalopathy and systemic manifestations (RVCLS) [36]. RVCLS is a rare disorder characterised by a progressive small-vessel disease that involves retina, brain, liver, and kidneys. Clinical features became manifest at different age, with an onset between 35 and 50 years. Generally, the migraine is one of the first neurological symptoms, followed by progressive cognitive and functional impairment [37].

Rare forms of cerebral amyloid angiopathy have been associated to the *ITM2B* gene, the so-called familial Danish and familial British dementia [38].

#### 2.1.4. Early Onset Familial Alzheimer Disease—EOFAD

Early-onset Alzheimer disease is a progressive dementia with the same phenotype as sporadic Alzheimer disease (AD) but with a presenile age of onset. Of all AD disease, almost 5% have a presenile onset and about 25% are familial [39]. Major genes recognised as responsible of familial forms of EOFAD are *PSEN1*, *APP,* and *PSEN2*. In particular, *PSEN1* mutations have been found in 20% to 70% of all EOFAD, *APP* mutations in 10% to 15%, and *PSEN2* mutations in about 5% [10,40]. Penetrance and clinical course of disease may vary according to gene involved. In particular, *PSEN1*-associated AD have typically an onset in the fourth–fifth decade (range from 30 y to 60 y), a rapid progression (less than 10 y), and associated features (seizures, myoclonus, language disturbance). *APP*-associated AD shows cerebral amyloid angiopathy and cerebral haemorrhage, with a progressive dementia that starts in the fourth–fifth decade (range from 30 y to 65 y). *PSEN2*-associated AD is a very rare disease, with a wide range of onset that may include senile presentations (40 y to 75 y). The duration of the disease is about 11 years. The penetrance is reduced, but not still estimated, because asymptomatic heterozygotes people with age >80 years have been reported [41,42].

Molecular mechanisms involved in the AD pathogenesis of *PSEN1*, *APP*, and *PSEN2* mutations are similar. PSEN genes are functionally involved in the proteolytic cleavage of amyloid precursor protein (*APP*) mediated by γ-secretases. Similarly, almost all pathogenic *APP* mutations involves the secretase sites, underlying the role of mutations in modifying *APP* processing. Although several efforts have been made in order to define genotype–phenotype relationship, to date no clear association has been proved [41,43].

#### 2.1.5. Frontotemporal Dementia

Frontotemporal dementias (FTD) are a group of cognitive disorders with predominant behavioural features at onset and atrophy of frontal and anterior temporal lobes. As well as for AD, autosomal dominant forms of FTD are generally early onset and show a positive family history of dementing disorder. About 80% of autosomal dominant FTD have been associated to mutations in three genes: *MAPT*, *GRN*, and *C9Orf72* [44]. Although there are some known histopathologic differences in brain of FTD patients according to involved gene, phenotypes are quite similar, and the genetic confirmation in vivo have to consider the investigation of a multigene panel.

*MAPT*-related FTD is characterised by a variable onset (from 40 y to 60 y), a rapid progression of disorder (duration of 5–10 y, occasionally 20–30 y), and a variable penetrance (mutation-related). The phenotype involves slowly progressive behavioural changes, language disturbance, and extrapyramidal signs. Less frequently patients may present parkinsonism, progressive supranuclear palsy, corticobasal degeneration, dementia with seizures [45].

*GRN*-related FTD shows an extreme variable onset (from 30 y to over 80 y) and rapid progression (from 3 to 12 y), with a reduced penetrance (about 90% at 75 y). Personality and behavioural changes are typical at onset, successively, language and movement disorders became manifest. In particular, the language phenotype is quite severe with progressive aphasia and semantic dementia. Movement disturbances involve parkinsonism, dystonia, and apraxia [45].

*C9Orf72*-related FTD have a wide phenotype spectrum, with a variable age at onset (30–70 y) and a quite full penetrance (about 100% at 80 y, with mutation-related changes). The correct evaluation of family history is complicated by the different phenotypes associated to *C9Orf72* mutations. In particular, the associated phenotype involves ALS, as well as FTD, with frequent overlap of these two disorders. Clinically, the *C9Orf72*-related FTD presents with behavioural changes, and executive or language disturbance, sometimes with features of parkinsonism (non tremorigen bradikynesia) [46].

Rare autosomal dominant forms of FTD have been associated to *VCP*, *FUS*, *CHMP2B*, and *TARDBP* genes. Unless for some distinctive histopathological feature, even for these rare forms there are not clinically features useful to predict the gene involved [44].

### 2.2. Phenotypes Due by Low Impact Variants or Genes (Multifactorial)

Numerous association studies allowed the identification of several susceptibility and risk alleles associated with the risk to develop dementing disorders [47,48]. Although some polymorphisms have been largely confirmed, other genetic variations lack a strong confirmation [49]. In genetic counselling, the evaluation of these polymorphisms must be carefully considered. The interpretation of susceptibility alleles for estimation of risk of disease is complicated and the potential associated risks must be carefully evaluated.

In this Section, main low impact genes associated to dementing disorders are summarised.

#### 2.2.1. Alzheimer Disease

Alzheimer disease is the most common neurodegenerative disorder. The typical onset of the disease is between the sixth and eighth decade, with a prevalence of <1% between 60 y and 69 y that increase to over 10% in 80 y people [50]. The histopathological features involve a neuronal loss in frontal, temporal, and parietal cortex, the formation of senile plaques composed by amyloid substance, and the neurofibrillar degeneration of neuronal cells. These brain changes can be occasionally observed in senile subjects but are very frequent in Alzheimer patients [51]. Clinically, patients show an initial mnemonic deficit that evolves in verbal and functional impairment (disorientation, amnesia, aphasia, apraxia, and agnosia). Successively, the movements became extremely slow, sometimes with myoclonus or choreoathetosis. The brain imaging (TC, MRI, 5-FDG PET) shows a whole brain atrophy [52,53,54]. Some studies reported that at least half of people with AD shows multiple brain modifications at autopsy [1]. In fact, it is frequent the coexistence of multiple dementing disorders (mixed dementia), that are often misdiagnosed in life due to the similarity of clinical phenotypes. Indeed, mnemonic symptoms are generally the most frequent presentation of different dementing disorders. Successively, AD patients can present behavioural changes (typically seen in FTD), poor judgment (typically seen in VaD), and sleep disturbance (typically seen in Lewy body disease). The overlapping of onset symptoms and the frequency of mixed dementia complicate the diagnosis.

It is estimated that one of four people over 55 years of age have a family history of dementia with at least one first-degree relative affected [44]. In AD, it is estimated that first-degree relative of AD patients had a lifetime risk of dementia about twice the risk of general population. This lifetime risk can slightly vary for different ethnical groups, but proportion between first-degree relatives and general population is conserved [44].

From a genetic point of view, the presence of E4 variant in *APOE* gene increases the risk for Alzheimer disease. Nevertheless, it is well known that the aetiology of senile Alzheimer disease (onset > 60–65 y) involves several genetic factors, so it is classified as a multifactorial disorder [5]. Although the diagnosis of AD may be supported by the presence of E4 genotype at the *APOE*, the typing is neither specific nor sensible analysis. In fact, the E4 allele increases the risk to develop AD, but it is no sufficient for developing disease. In particular, it is estimated that the E4 allele confers an AD risk of about 10–20% if in heterozygous state and 25–30% if in homozygous state [55]. On the contrary, about 40% of AD patients do not carry an E4 allele. Therefore, the *APOE* genotyping can just support the diagnostic path of AD, but it is not useful to confirm not to exclude the diagnosis.

In reason of the association between *APOE* genotypes and AD, several studies tried to identify allele-specific molecular treatments. *APOE* shows pleiotropic functions in CNS, and treatments focused on *APOE* action have to consider these numerous roles. Although several molecular approaches have been developed to modify the impact of *APOE4* genotype on brain, to date no approved treatment have been introduced in the clinical practice. Therapeutic agents that have been studied in murine models and in humans are generally involved in the *APOE* lipidation and Aβ clearance (as RXR, LXR, PPARγ agonists), in modulation of *APOE*-connected neuro-defensive effects, and in leakage of blood-derived toxic molecules in brain (as cyclosporine A) [56]. To date, the ClinicalTrials database lists 118 ongoing interventional and 53 observational studies on AD patients that involve *APOE* genotyping [57]. The collection of *APOE* genotype for patients enrolled underlies the increased role of *APOE* alleles in definition of pathological trajectories in AD. The evaluation of *APOE* genotypes together with pharmacokinetic and pharmacodynamic data in the development of new therapeutic agents permits to evaluate possible molecular influences of *APOE* alleles. Although the great diffusion of *APOE* genotype in diagnostic path, no useful pharmacologic differences have been demonstrated for ε2/3/4 haplotype carriers. As well as for other neurodegenerative diseases, pharmacogenetic tests used in clinical practice involve the CYP metabolisms to determine the metabolisation profile.

To date, more than 40 loci [58] and rare genetic variants [59] have been recognised as susceptibility factors for AD. Nevertheless, the *APOE* is the unique factor which genotyping is useful in the diagnostic path of AD. Other factors are not able to significantly modify the risk of disorder or the therapeutical approach. In this scenario, the familial risk calculated on the presence of one or more familial members suffering of AD is predominant and cannot be significantly modified by susceptibility alleles [60].

#### 2.2.2. Vascular Dementia

Vascular dementia (VaD) is a neurodegenerative disorder in which the cognitive impairment is secondary to cerebral vascular damage. Vascular dementia is the second most common dementing disease worldwide. Most of cases of VaD are sporadic and recognise a multifactorial pathogenesis. Several environmental factors have been associated to a different susceptibility to VaD (lifestyle, comorbidities, hypertension, dyslipidemia, etc.) [61,62]. To date, several association studies and meta-analyses have been performed in order to identify genetic susceptibility factors involved in VaD. Genes frequently reported in association to VaD are classically related to inflammation (*APOE*, *MTHFR*, *TGFB1*, *TNF*) [63,64]. Nevertheless, a strong confirmation of association is lacking. On the other hand, quantitative association studies have revealed different serum or CSF levels of TGFB1, TNF, IL1B, and IL6 in VaD patients, when compared with controls [47]. Furthermore, the clinical heterogeneity of VaD suggest the existence of different genetic risk profiles and many molecular pathogenetic pathways. In this scenario, it is not surprising that different haplotype at *APOE* gene have been associated with different molecular mechanisms of vascular damage. In particular, E2 haplotype is associated with hemorrhages, E4 haplotype with β-amyloid accumulation [47,65] Lack of strong confirmation data in genetic susceptibility of VaD seem to support a combined effect of genetic variants and environmental factors. Given the high frequency of dementia in general population, with about 25% of people having at least one family member suffering from dementia, the recurrence risk for first-degree relatives of VaD patients is difficult to estimate. However, in order to perform differential diagnosis between multifactorial and mendelian forms of VaD, a full familial evaluation should always be performed. Furthermore, a risk estimation for relatives of VaD patients should take into consideration all environmental factors (cardiovascular, thrombotic, metabolic, etc.) [66].

Neurodegenerative disorders are often presenting in simultaneous times. In the elderly, in fact, mixed pathologies of the brain are common, and their prevalence increases with aging [47,67]. In this complex scenario, it is not surprising that genetic factors play a synergistic role, with several types of influences on the etiopathogenesis. Genetic pleiotropy is largely recognised in the development of neurodegenerative disorders. The main example is the *APOE* gene, that has been involved in almost all cognitive disorders. Furthermore, several GWAS revealed that different dementing disorders share same risk loci [68,69].

#### 2.2.3. Frontotemporal Dementia

Frontotemporal dementia (FTD) is a common presenile dementia with predominant behavioural features at onset and atrophy of frontal and anterior temporal lobes [70]. It is estimated that about 30–50% of FTD have a genetic basis, with 10–30% due to known genetic high impact variant [71]. Clinically, there are five main phenotypes recognised in FTD: behavioural variant, PPA (primary progressive aphasia), semantic dementia, FTD with parkinsonism, and FTD-MND (FTD with motoneuron disease). Interestingly, the positive family history is more frequent in patients with behavioural variant of FTD than in patients with PPA [72]. These data support the existence of a strong genotype-phenotype relationship in FTD [45,73]. Unfortunately, a strong correspondence has been just revealed between gene and histopathological features. Clinical phenotypes are often overlapping between different forms, in fact molecular analyses should evaluate a panel of known genes to improve the diagnostic yield of genetic test. Due to the phenotype-specific distribution of familial forms of FTD, the estimated empirical recurrence-risk for first-degree relatives of affected patients is about 20% [44,74]. This estimated risk is about twice the risk of dementia in general population. It is important to perform a complete evaluation of family history, in order to distinguish familial from sporadic forms. In fact, the estimation of recurrence risk may vary from 20% for sporadic forms to 30–50% for familial FTD. Nonetheless, about 5% of sporadic FTD shows mutation in known FTD-genes [75]. As a consequence, the genetic evaluation of FTD should be considered even in sporadic cases, particularly in patients with an incomplete family history (adoption, premature death for accidental events, etc.).

## 3. Genetic Counselling

The extreme diffusion of dementing disease in the population complicates the application of appropriate genetic counselling protocols. Actually, the geneticist is called for evaluation in cases with a known family history of disease or in selected phenotypes (early onset, atypical or syndromic presentation, etc.). As a consequence, questions from familial members of an affected patients often remains unsolved.

Notably, “Genetic counseling is a communication process which deals with the human problems associated with the occurrence, or the risk of occurrence, of a genetic disorder in a family” [76]. As largely known, dementing disorders recognise several genetic factors in their aetiopathogenesis. Accordingly, an accurate genetic counselling should be offered for all patients suffering from dementing disorder. The genetic counselling will help patients and their families in understand the disease, the risk of occurrence, and possible strategies to control it. Furthermore, the geneticist evaluates the useful of genetic analyses in the diagnostic path, in order to better define the diagnosis and the sub-phenotype of the dementing disease. In this scenario, the identification of genetic variants will support the administration of therapeutical protocols improving the effectiveness of treatments. In our opinion, a genetic counselling should be considered for all patients that receive a diagnosis of dementing disorder. The geneticist evaluation will help not only patients and their family members, but also neurologists in order to identify high and low impact genetic variants useful for administration of personalised medicine protocols [77,78].

Despite frequently suggested, a multidisciplinary approach to the dementing patient is very rare in clinical practice. Numerous genetic counselling protocols have been developed for neurodegenerative and dementing disorders [39,79,80,81,82,83,84]. Generally, counselling protocols are diagnosis-specific [39,80,81,83,84] and are focused on the genetic test (pre--symptomatic/diagnostic) and their implications [83,84,85]. To our knowledge this is the first protocol that involves the geneticist in the diagnostic path of neurodegenerative disorder. Many neurodegenerative disorders share some onset features that make the diagnosis challenging. Given the importance of an early diagnosis through a complete evaluation of patient (familial, clinical, and instrumental), the diagnostic path should be performed by a multidisciplinary team of medical doctors (neurologist, cardiologist, radiologist, geneticist) [86]. In this scenario, the genetic counselling can be performed before the clinical diagnosis and steps of counselling should consider all different possible diagnosis. Previously reported genetic counselling protocols are very useful for patients and families with a known neurological diagnosis or a known pathogenetic mutation that segregates in the family. The counselling protocol here described extends the involvement of geneticist to the first phases of diagnosis, without modifying the available and largely used protocols for genetic diagnostic or pre-symptomatic test for neurodegenerative and dementing disorders.

**Figure 1:**
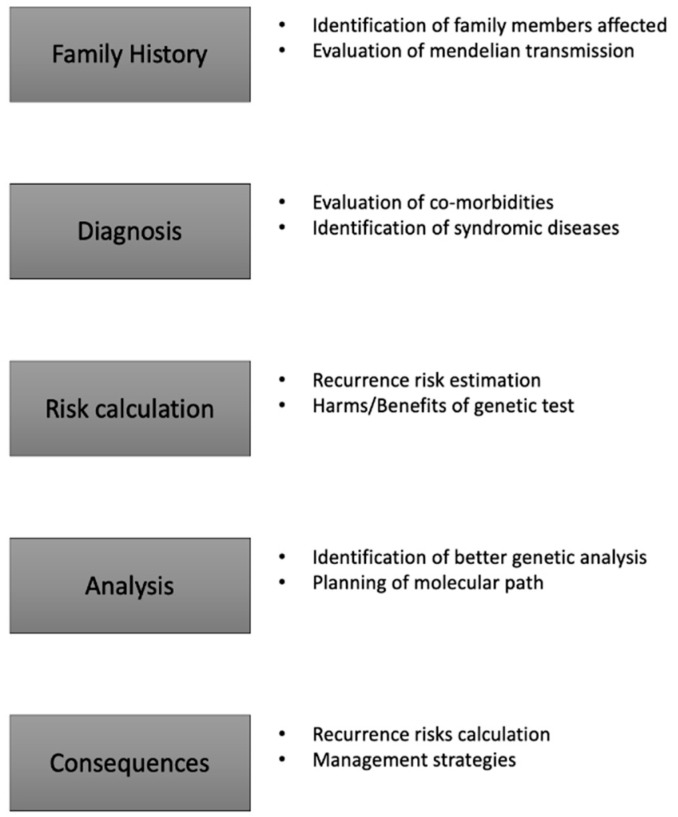
Steps in genetic counselling for neurodegenerative disorders.

In order to define essential steps to consider in genetic counselling, we will report a point-by-point list (summarised in Figure 1).

I. Evaluation of family history. Geneticist collects an accurate (at least three-generational) pedigree of the family, with details about neurological disorders in the family, age at death, consanguinity. It is important to ask the right questions. In particular, in order to evaluate even the subclinical (or undiagnosed) dementing disorders, the geneticist should ask about ability to remember of familial members. The counselling process should stimulate memories of events. This process is aimed to identify possibly undiagnosed dementing disorders in the family. The full pedigree that can be collected after a complete evaluation of the family will permit to evaluate mendelian inheritance pattern useful for risk calculation and for administration of genetic tests.

II. Evaluation of diagnosis. Medical geneticist performs an evaluation of clinical, laboratory, and instrumental evaluations conducted on the patients. The accurate definition of the phenotype permits to identify possible sub-phenotypes of disease and possible non-correlated disorders. In particular, it is known that an early onset of AD is often associated to variants in *PSEN1* and *PSEN2* genes. Furthermore, the presence of familial members with ALS can suggest an expansion in *C9Orf72*. Nonetheless, a phenotype in which dementia is presenting with ataxia or with dystonia may suggest the evaluation of dementing spinocerebellar ataxias (as SCA2) or brain accumulation disorders (as Fabry disease).

III. Risk evaluation and communication. The evaluation of full pedigree and phenotype permits to identify the possible genetic burden of the disorder. Basing on these evaluations, geneticist should anticipate estimated recurrence risks for the disorders and potentiality of genetic test. In this scenario, it is important the communication of pre-test risks and the implication of a positive or negative test result.

IV. Identification of possible genetic analyses. Considering the pedigree and phenotype, the appropriateness and usefulness of genetic analysis have to be evaluated. In particular, an accurate definition of genetic analysis to perform on patient should be conducted according to clinical, therapeutical, and economic implications.

V. Information about details of genetic test. In order to conduct an appropriate pre-test genetic counselling, geneticist should inform the patient and their family of characteristics, limits, and possible results. The extension of the communication process to familial members is particularly important in dementing disorders. It is well known in fact that sometimes dementing disorders compare with psychiatric features and patients may give their informed consent and successively forget it. For these reason, genetic counselling should involve at least one familial member, useful to collect a complete family history, but also to record the molecular diagnostic protocol and the informed consent procedures. Furthermore, geneticist informs patients and family members about potential harms and benefits of the genetic analysis. In this context, a non-directive counselling ensures informed and aware decisions about genetic test.

VI. Post-test genetic counselling. Results of genetic analyses have to be explained in a post-test genetic counselling. Topics explained in the pre-test genetic counselling have to be further considered in the post-test counselling. In particular, geneticist communicates the corrected estimation of recurrence risk and the available therapeutical strategies. If available, strategies to prevent the recurrence of disease should be appropriately communicated (pre-implantation genetic diagnosis, prenatal testing, etc.).

In order to perform an exhaustive genetic counselling, benefits and harms potentially associated to the genetic test have to be considered. Medical, psychological, and social issues can modify benefits and harms, that should be personalised according to propositus. Table 1 summarises the main medical and psychosocial benefits or harm to consider in genetic counselling.

Furthermore, the analysis strategy should be designed according to clinical diagnosis, frequency/type of genetic variants, and economic burden of analyses. In this scenario, it is important to communicate the analytical protocol to the patient, that should be informed about limits of genetic tests. For example, the evaluation of a FTD can involve the genotyping of expansion in *C9Orf72* and the sequencing of associated genes (*MAPT*, *GRN*, *VCP*, *FUS*, *CHMP2B*, and *TARDBP*). People without mutations in this FTD-specific genes can be re-evaluated, and other genes can be tested. A second-level analysis can identify mutations in other genes [75], improve the diagnostic yield of genetic test, and complete the molecular evaluation of patient. As expected, the time of testing can vary according to the number of analyses performed. The counselor should anticipate the analytical protocol to the patient, in order to avoid the false sense of security derived from a first-level negative (wild-type) result.

## 4. Burden of Variants of Uncertain Significance in Dementing Genes

The availability of high throughput technologies for exome sequencing have improved the time for molecular diagnosis of genetic dementing disorders. Otherwise, the time required to perform an accurate analysis of big sequencing data is critically related to the accuracy of phenotype. In this scenario, the clinical genetic evaluation of patients consents to define phenotype and to select potentially associated genes. Nevertheless, a big sequencing analysis detects a great number of genetic variants of uncertain significance (VUS) that requires further analyses in order to be correctly classified as benign or pathogenic. In order to estimate how deep is the knowledge about dementing genes, GnomAD database has been interrogated [88]. In Table 2, we summarised all GnomAD variants in a selection of dementing genes. Of all variants, rare coding variants has been selected and divided in variants with and without a ClinVar classification. As expected, the amount of known rare variants is very low with an average percentage of ClinVar classified variants of 8.3%. Considering the high number of VUS found in healthy people, an analysis in affected patients will revealed at least a similar amount of VUS. The re-evaluation of these rare variants can take a very long time, that can be improved by prioritisation algorithms based on clinical and familial data. In particular, the genetic counselling and the neurological evaluation permit to characterise the patient phenotype and transmission model of inheritance. Sub-phenotypes detected by clinical and instrumental patient evaluation can help in prioritising genes to be evaluated. Furthermore, an accurate pedigree evaluation may suggest a transmission model of inheritance and consequently the evaluation of a gene subset associated with this inheritance model. As reported, the re-classification of unknown rare genetic variants in neurological disorders can improve the diagnostic yield of genetic test from 21% to about 30% [89].

## 5. Discussion

Dementing disorders recognise several types of genetic factors that influence onset, progression, and transmission of disease. Sometimes, as seen, genetic variants can help in the definition of therapeutical strategies, suggesting pharmacological prevention (CADASIL) or surgery intervention (Fabry disease). Recently, the improvement in technological strategies for DNA sequencing make possible the simultaneous evaluation of several genes in a small time. Nevertheless, the indiscriminate application of extended genetic analysis determines an unsustainable economic burden for genetic laboratories. Furthermore, the extended genetic analysis requires times not compatible with a diagnostic process. Genetic counselling provides familial and phenotypic information that could help in prioritising the genetic analyses. In particular, the next-generation genetic analysis should first consider genes most probably involved in the patient’s phenotype, secondary a full exome evaluation can be performed on genes associated to less-fitting phenotypes. As reported, a gene-panel approach is a rapid and useful strategy yet applied to other disorders that recognised different genetic etiologies [86,90]. In neurodegenerative disorders, a recent study reported an interesting diagnostic yield for exome sequencing, with an overall diagnostic yield of 21% (spanning from 40% for spastic paraparesis to 8% for dystonia). In particular, despite the small sample size, the study reported a diagnostic yield of 10% for dementia, that can be improved by an accurate bioinformatic analysis and further characterisation of potentially damaging genetic variants [89]. As largely reported, the NGS analysis provide a great number of genetic variants that require a further classification on clinical, molecular, and familial data. Polygenic disorders like neurodegenerative diseases can benefit from whole exome analysis or target sequencing in order to complete the diagnostic path and identify mutations associated to the phenotype [91]. As reported in similar disorders, the automatic classification of genetic variants [92] can be improved applying a prioritisation algorithm [93]. The correct design of rules included in the prioritisation algorithm can potentially permit the reclassification of about 70% of VUS identified [93]. In the neurological context, the known phenotypic and genetic heterogeneity improves the number of putative genes to be investigated for molecular diagnosis. Furthermore, the high amount of expected VUS in neurological genes complicates the interpretation of genetic analysis, that often require a deep clinical re-evaluation. In this context, the whole application of genetic counselling in neurological characterisation of dementing disorders will provide a full phenotype dataset, that can support the interpretation and classification of genetic data. In particular, the complete evaluation of genetic variants throughout ACMG criteria, phenotype, and pedigree data will improve time and cost for genetic test in neurodegenerative disorders [89]. Further studies, with evaluation of a large number of neurological patients, will be required in order to evaluate the exact improvement of genetic counselling in the diagnostic path of patients. To date, some small studies reported interesting results, with an average diagnostic yield between 10% and 30% [89,94].

Furthermore, the overall application of genetic counselling to patients suffering from dementing disorders will improve not only the planning and administration of analytical molecular protocols but will also reduce time for diagnosis. In clinical practice, the rarity of medical doctors specialised in genetics can now be solved by the application of telemedicine. The on-line genetic counselling, yet applied for professional consultation, can now be easily introduced in clinical practice and extended to patients. Recently, the global pandemic of COVID-19 and the necessary precautions to avoid infection diffusion have determined the translation of several medical procedures in novel delivery modes, with the development of telemedicine [95,96]. As reported, the introduction of genetic counselling from the first phases of diagnosis can complete the evaluation improving the time for diagnosis. To date, the diffusion of telemedicine protocols can support the diffusion of genetic counselling in neurology. The diagnostic path, the planning and administration of molecular analytical protocols, and the evaluation of genetic results should be performed by a geneticist [85] and not only suggested, as frequently due in many medical centres. The diagnostic and therapeutical protocols for dementing disorders should provide all necessary clinical and instrumental evaluations. In clinical practice sometimes the immediate unavailability of professionals prolongs the diagnostic odyssey because patients have to plan on their own the clinical and instrumental evaluations required. It is important that every professional and every diagnostic medical centre with expertise in neurodegenerative dementing disorders dispose of a network of professionals in order to plan each medical protocol useful in the diagnostic path. In this scenario, the application of telemedicine for genetic consultation should be recommended to simplify its introduction in clinical practice.

The clinical complexity of neurodegenerative disorders, the frequency of co-morbidities, and the high phenotypic heterogeneity of genetic disease complicate the selection of patients eligible for genetic analysis. In this context, the overall application of exome sequencing is likely to be non-economically sustainable for the health care systems. It is particularly important considering the increasing prevalence of neurodegenerative disorders in elderly, with about 43,000 people suffering from dementia in Europe (60–70% AD, 15–20% VaD) and an incidence rate that increase from 2.4 per 1000 (person/year) in 65–69 years older to 70.2 per 1000 in over 90 years older [50]. Considering the economic and demographic scenario, the application of genetic counselling will help in selection of patients eligible for genetic analysis. As seen, an accurate familial and phenotypic characterisation can support the identification of rare phenotypes and suggest the genetic analysis. The diagnostic odyssey is a frequent report in neurodegenerative disorders. The smart application of exome analysis to dementia patients, with a pre-analytical selection on familial, clinical, and instrumental features, can improve the diagnostic yield of genetic test and reduce diagnostic times. Furthermore, the impact on management of neurodegenerative disorder may be improved in about two thirds of diagnosed patients [89].

## Figures and Tables

**Table 1:** Potential benefits and harms of genetic testing for neurodegenerative disorders (modified from [87]).

Category	Benefits	Harms
Medical issues	Preventive and/or therapeutic interventions	ineffective or harmful preventive or therapeutic interventions
	increased surveillance	incidentalomas
	avoiding unnecessary surveillance	
	refinement of prognosis	
	clarification of diagnosis	
Psychosocial issues	reduction of uncertainty	increased anxiety and guilt
	opportunity of psychological adjustment	
	alerting other family members to genetic risk	

**Table 2:** Burden of rare genetic variants in dementing genes. For each gene, the table provide: total number of described variants in GnomAD database (3rd column), total rare coding variants (coding variants with allele count < 5) and rare coding variants described in ClinVar international database (4th column), percentage of rare variants not listed in ClinVar database (5th column).

Gene	OMIM	GnomAD Variants	Rare Coding Variants (Reported in ClinVar)	Percentage of Non-ClinVar Variants
*TARDBP*	*605078	479	112 (14)	87.5
*PSEN2*	*600759	954	288 (15)	94.8
*VCP*	*601023	928	185 (29)	84.3
*PSEN1*	*104311	631	234 (21)	91.0
*FUS*	*137070	1211	289 (24)	91.7
*GRN*	*138945	982	387 (42)	89.1
*MAPT*	*157140	1181	423 (23)	94.6
*APP*	*104760	1381	415 (19)	95.4
*HTRA1*	*602194	734	238 (20)	91.6
*DCTN1*	*601143	2246	686 (118)	82.8
*COL4A1*	*120130	3112	757 (34)	95.5
*COL4A2*	*120090	3049	909 (36)	96.0
*TUBA4A*	*191110	436	141 (4)	97.2
*CHMP2B*	*609512	355	133 (4)	97.0
*MATR3*	*164015	1064	350 (19)	94.6
*CSF1R*	*164770	1627	482 (14)	97.1
*SQSTM1*	*601530	992	353 (49)	86.1
*GBA*	*606463	756	262 (42)	84.0
*TREX1*	*606609	457	240 (31)	87.1
*NHLRC1*	*608072	401	216 (36)	83.3
*EPM2A*	*607566	564	202 (45)	77.7
*OPTN*	*602432	996	347 (21)	93.9
*ANXA11*	*602572	1138	371 (1)	99.7
*HNRNPA1*	*164017	830	173 (3)	98.3
*TBK1*	*604834	979	87 (22)	74.7
*ITM2B*	*603904	379	130 (0)	100.0
*GSN*	*137350	1461	467 (9)	98.1
*CST3*	*604312	274	97 (1)	99.0
*ANG*	*105850	182	84 (5)	94.0
*CCNF*	*600227	1485	426 (2)	99.5
*DNMT1*	*126375	2636	661 (102)	84.6
*DNAJC5*	*611203	343	79 (16)	79.7
*NOTCH3*	*600276	2928	1783 (73)	95.9
*PRNP*	*176640	251	130 (10)	92.3
*TYROBP*	*604142	332	91 (4)	95.6
*UBQLN2*	*300264	349	176 (8)	95.5
*PLA2G6*	*603604	1554	480 (32)	93.3
*COASY*	*609855	957	392 (5)	98.7
*C19orf12*	*614297	312	92 (11)	88.0
*FTL*	*134790	368	139 (11)	92.1
*PANK2*	*606157	893	345 (31)	91.0
*WDR45*	*300526	461	144 (18)	87.5

## Data Availability

The data generated in the present study are included within the manuscript.
